# A Markerless 3D Computerized Motion Capture System Incorporating a Skeleton Model for Monkeys

**DOI:** 10.1371/journal.pone.0166154

**Published:** 2016-11-03

**Authors:** Tomoya Nakamura, Jumpei Matsumoto, Hiroshi Nishimaru, Rafael Vieira Bretas, Yusaku Takamura, Etsuro Hori, Taketoshi Ono, Hisao Nishijo

**Affiliations:** 1 System Emotional Science, Graduate School of Medicine and Pharmaceutical Sciences, University of Toyama, Toyama, 930–0194, Japan; 2 Department of Anatomy, Graduate School of Medicine and Pharmaceutical Sciences, University of Toyama, Toyama, 930–0194, Japan; 3 Behavioral Science, Graduate School of Medicine and Pharmaceutical Sciences, University of Toyama, Toyama, 930–0194, Japan; University of Münster, GERMANY

## Abstract

In this study, we propose a novel markerless motion capture system (MCS) for monkeys, in which 3D surface images of monkeys were reconstructed by integrating data from four depth cameras, and a skeleton model of the monkey was fitted onto 3D images of monkeys in each frame of the video. To validate the MCS, first, estimated 3D positions of body parts were compared between the 3D MCS-assisted estimation and manual estimation based on visual inspection when a monkey performed a shuttling behavior in which it had to avoid obstacles in various positions. The mean estimation error of the positions of body parts (3–14 cm) and of head rotation (35–43°) between the 3D MCS-assisted and manual estimation were comparable to the errors between two different experimenters performing manual estimation. Furthermore, the MCS could identify specific monkey actions, and there was no false positive nor false negative detection of actions compared with those in manual estimation. Second, to check the reproducibility of MCS-assisted estimation, the same analyses of the above experiments were repeated by a different user. The estimation errors of positions of most body parts between the two experimenters were significantly smaller in the MCS-assisted estimation than in the manual estimation. Third, effects of methamphetamine (MAP) administration on the spontaneous behaviors of four monkeys were analyzed using the MCS. MAP significantly increased head movements, tended to decrease locomotion speed, and had no significant effect on total path length. The results were comparable to previous human clinical data. Furthermore, estimated data following MAP injection (total path length, walking speed, and speed of head rotation) correlated significantly between the two experimenters in the MCS-assisted estimation (r = 0.863 to 0.999). The results suggest that the presented MCS in monkeys is useful in investigating neural mechanisms underlying various psychiatric disorders and developing pharmacological interventions.

## Introduction

Patients in various psychiatric disorders, such as autism and schizophrenia, display emotional and social deficits [1,2]. Primate animal models of these diseases are necessary to investigate the neural mechanisms underlying these disorders and develop pharmacological interventions, because of the many similarities between monkeys and humans in brain morphology, cognitive skills, and social complexity, as well as substantial differences between primates and rodents in these points [3–9]. Thus, primate animal models have strong face and construct validity for investigating the neural mechanisms underlying emotional and social deficits in humans [9].

Since the motion of body parts and postures in animal behaviors can reflect their emotions, intentions, and ongoing goals, extensive studies have analyzed the motion and posture of monkeys to score their emotional responses to stimuli (e.g., [10–14,15]) and social interactions (e.g., [5,10,16–18]). Most of these studies manually scored behaviors through the visual inspection of videos. However, results based on visual inspection may vary because of differences in experience, skill, and sensitivity to behaviors between experimenters, which may decrease the reproducibility of the results [19].

Digital motion capture of major body parts can quantify motions and postures of animals in more reproducible ways than visual inspection [19]. Motion capture systems (MCS) have been used in many previous studies in monkeys for investigating motor functions [20–23]. Most previous studies used analytical systems requiring markers attached to body parts in order to track positions. However, application of the markers themselves is stressful and could distract or distress monkeys [24–26]. These findings strongly suggest that a markerless MCS is more appropriate to analyze emotional states and social interactions in monkeys, which could be easily altered by stress.

We previously proposed a markerless 3D digital MCS for rodents [27]. The MCS can represent the 3D motion of the trunk and head, and quantify various behaviors [19,27]. However, primate behaviors are highly complex when compared with rodents, since primates have relatively longer extremities with joints that have higher degrees of freedom. In this study, we propose a novel markerless MCS by improving the previous fitting algorithm applied to rodents [27] so that it can represent the main joints of the body in large animals including monkeys. To validate the MCS, first we recorded sample data of monkeys performing a shuttling behavior, in which, to induce various postures in the monkeys, an obstacle was introduced in various positions that they had to avoid. We then compared the 3D positions of the body parts estimated by the MCS with those estimated manually by experimenters. Second, to confirm the reproducibility of the results by the MCS, the same analyses of the above experiments were repeated by a different user, and the differences between the users were examined. Third, to demonstrate the effectiveness of the MCS for quantifying emotional states, we analyzed the effects of acute methamphetamine (MAP) administration on spontaneous behaviors using the MCS. Injection of MAP has been reported to induce schizophrenic like symptoms in monkeys as well as humans [28, 29].

## Materials and Methods

### Subjects

Four adult males and one adult female monkey (*Macaca fuscata*) were used in this study. The monkeys were housed in individual home cages on a 12 hr on/12 hr off lighting schedule with food and water available *ad libitum*. The sizes of the home cages used in the present study were consistent with the criteria of the cage sizes for monkeys in the National Institutes of Health guide for the care and use of laboratory animals 8th edition. Supplemental fruit and vegetables were given after each day’s testing session. To check the monkey’s health, their weight was routinely monitored and their physical size and feces were monitored every day by animal care staffs and experimenters under the supervision of a veterinarian. The criteria to terminate the experiment was that the body weight of subjects became less than 85% of the pre-study original, and that subjects showed any signs of suffered states and/or vomiting after MAP administration. The monkeys were treated in strict compliance with the United States Public Health Service Policy on Humane Care and Use of Laboratory Animals, the National Institutes of Health Guide for the Care and Use of Laboratory Animals, and the Guidelines for the Care and Use of Laboratory Animals of the University of Toyama. This study was approved by the Committee for Animal Experiments and Ethics at the University of Toyama (Permit Number: A2016med-8). Environmental enrichment, in the form of toys, was provided daily, and all efforts were made to maximize the well-being of the animals.

### Experimental setup

**Fig 1:**
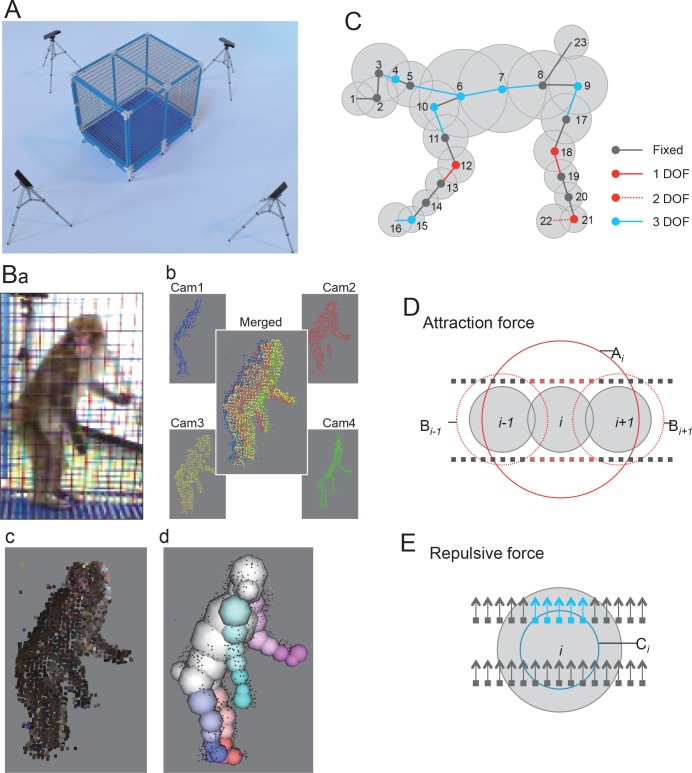
Markerless MCS for monkeys. A: Experimental setup consisting of a monkey cage with four depth cameras. B: Schematic illustration of processing steps of the present MCS. A monkey was captured by four depth cameras (Cam1-4) (a, b), and the images were merged to make a 3D image of the monkey represented by 3D points on the entire surface of the monkey (b). Simultaneously captured color images were mapped onto the 3D points (c). Finally, a skeleton model of the monkey was fitted onto the 3D image (d). C: A skeletal model of a monkey used in the present study. The model consisted of spheres connected by joints. Centers of the spheres, where lines are connected, indicates joints. Number of degrees of freedom (DOF) in each joint is shown by color. D: Attraction force from the points. Small squares represent captured 3D points. Gray spheres represent spheres in the model. The red points attract the sphere *i*. E: Repulsive force from the points. Arrows indicate the surface normal at the points. The blue points push the sphere *i* away. Other descriptions are same as D.

Fig 1A shows the experimental setup used in this study. A monkey was put in the recording cage and its behavior was captured using Kinect sensors (Kinect for Windows ver. 1, Microsoft) placed around the cage at the 3, 6, 9, and 12 o’clock positions. The Kinects were connected to a PC (Core i7 [4 cores], 8GB memory). The distance between each Kinect and the center of cage was 1.6 or 1.9 m and the Kinects were positioned 1.1 m high above the floor. The Kinects were tilted at an angle of 10° below the horizontal plane.

### Markerless 3D motion capture

We developed a markerless 3D MCS for a freely-moving monkey by extending our previous MCS used in rats [19,27]. The present MCS captured the motion of a monkey by acquiring 3D video of the monkey and by fitting a skeleton model of the monkey onto a 3D image of the monkey in each frame of the video (Fig 1Ba-b). The 3D video was acquired by the same method as the previous study [19]. Each frame of the 3D video was represented by 3D points on the surface of the monkey. Briefly, the 3D points were acquired by four depth cameras, each of which was embedded in a Kinect sensor. A depth camera captured a depth image, in which each pixel represented the distance from the camera to the surface of the monkey. A depth image could then be easily converted into 3D points on the surface of the object. To cover the entire surface of the object, the 3D points captured by the four depth cameras from different viewpoints were integrated (Fig 1Bb). Furthermore, the color of each of the point was obtained by mapping color images simultaneously captured by color cameras embedded in the same Kinect sensor (Fig 1Bc). In addition, the surface normal at each point was calculated. Points on background objects (i.e., objects except for the monkey) were ignored by excluding and including points within user-defined regions in the 3D space.

A 3D image captured by the above method (e.g., Fig 1Bc) represents a 3D “hull” of a monkey. The fitting algorithm works as if it physically houses the skeleton model into the 3D hull (Fig 1Bd). A similar physics-based algorithm was used in our previous MCS for rats [19]. Fig 1C shows the skeleton model that we used in the present study. The right limbs are not displayed for simplicity. The model consisted of 23 spheres (Fig 1C; 1–23) connected with joints rotating within different ranges corresponding to the anatomical constraints of the joints. The dimensions of the model were changed for each subject, while the range of the joints’ rotation were constant across subjects. The model was fitted onto the 3D hull with the aid of Bullet Physics Library (ver. 2.8.1, an open-source physics engine, http://bulletphysics.org/) through the following physics simulation. In the simulation, the following three types of forces from the 3D points to the spheres of the model were assumed to make the model converge within the 3D hull of the monkey. First, attraction forces were assumed to lead the model into the hull (Fig 1D). The value and direction of the attraction force for each sphere *i* ($\vec{f_{ai}}$) was calculated using the following equation that was applied to the center of the sphere:

$$\vec{f_{ai}}=\alpha m_{i}\sum_{j}^{n}a_{j}\vec{S_{i}P_{j}}$$

where *α* is a constant, *m*_*i*_ is the weight of sphere *i*, *S*_*i*_ is the center of sphere *i*, *P*_*j*_ is the position of the 3D point *j* (*j = 1*, *…*, *n*), and *a*_*j*_ is 1 if *P*_*j*_ is in the region defined by the following equation:

$$\begin{array}{c} k\neq i\\ A_{i}\cap {\left(B_{k}\right)}^{c} \end{array}$$

where *A*_*i*_ is a larger spherical region around *S*_*i*_, while *B*_*k*_ is the smaller region around *S*_*k*_ (Fig 1D), otherwise *a*_*j*_ is 0. Thus, each of the spheres of the model was attracted to the points around the sphere that are not near other spheres. Second, repulsive forces were assumed to keep the model within the hull (Fig 1E). The value and direction of the repulsive force for each sphere *i* ($\vec{f_{ri}}$) was calculated using the following equation that was applied to the center of the sphere:

$$\vec{f_{ri}}=\beta m_{i}\sum_{j}^{n}r_{j}\vec{S_{i}P_{j}}/\left\|\vec{S_{i}P_{j}}\right\|$$

where *β* is a constant and *r*_*j*_ is 1 if point *j* fulfills the following equations:

$$P_{j}\in C_{i},\vec{N_{j}}∙\vec{S_{i}P_{j}}>0$$

where *C*_*i*_ is a spherical region around *S*_*i*_ (Fig 1E) and $\vec{N_{j}}$ is the surface normal of point *j*, otherwise *r*_*j*_ is 0. Thus, each of the spheres of the model was pushed away from each point near the sphere, when the sphere is inside the surface around the point. Third, since the faces of our monkeys were redder than most of the other parts of their bodies, to make the direction of the head of the model more accurate, the following attraction force ($\vec{f_{ah}}$) towards the jaw (sphere 1 in Fig 1C) was assumed:

$$\vec{f_{ah}}=\gamma m_{1}\sum_{j}^{n}f_{j}\vec{S_{1}P_{j}}$$

where γ is a constant and *f*_*j*_ is 1 if point *j* fulfills the following equations:

$$H_{min}\leq h_{j}\leq H_{max},S_{min}\leq s_{j},V_{min}\leq v_{j},P_{j}\in A_{3}$$

where *H*_*min*_, *H*_*max*_, *S*_*min*_, and *V*_*min*_ are constant, *h*_*j*_, *s*_*j*_, and *v*_*j*_ are the hue, saturation, and value of the color of point *j*, respectively, and *A*_*3*_ is the spherical region around the head (sphere 3 in Fig 1C), otherwise *f*_*j*_ is 0. Thus, the sphere corresponding to the jaw was attracted by the red colored points around the head to represent the head direction more accurately. The physics simulation was continued until the simulated physical system reached a steady state in which all sphere shifts in the model fell below a small value.

With the fitting algorithm, the motions of the monkey in a 3D video were semi-automatically estimated offline as follows (see also S1 Movie). In the first frame of the video, the model was manually located near the 3D hull of the monkey, and the model was fitted onto the 3D hull by the physics-based algorithm described above. Then, the resultant positions of the spheres in the model were recorded and used as the initial position for the next frame. The process was repeated until the final frame of the video. An experimenter observed throughout the processing. When the positions estimated by the fitting algorithm were significantly wrong (e.g., the left and right limb were confused), the experimenter paused the processing, manually corrected the positions, and restarted the process from the corrected frame. As a result, the trace of each of the spheres in the model throughout the video recording was acquired. Finally, the traces were filtered with a loess filter (time window: 0.5 sec) using the ‘‘smooth()” function of Matlab, version R2013b.

Software for the pose estimation using a 3D video, its source codes, and a set of sample data files are provided in S1 File.

### Validation of the MCS

**Fig 2:**
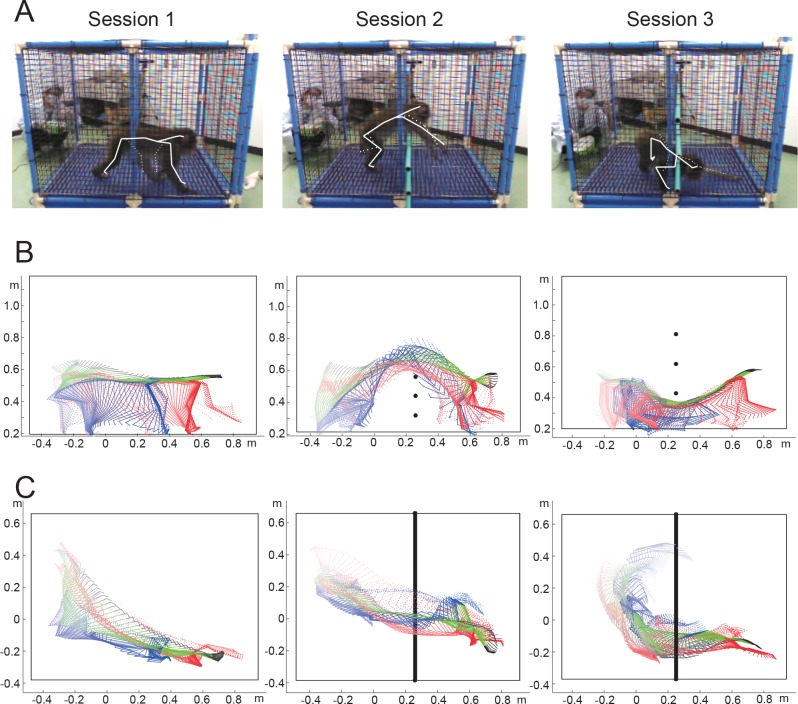
Examples of captured motion in the shuttling task. A: Snapshots of the video captured in the task without obstacles (session 1), the task with obstructing bars at a low height in the middle of the cage (session 2), and the task with obstructing bars at a medium height in the middle of the cage (session 3). White solid lines indicate inner skeletons in the trunk and right limbs, dotted lines indicate inner skeletons in the left limbs. B and C: Traces of the estimated posture from the side view (B) and top view (C) based on the snapshots shown in A. Black bars and points represent the obstacle bars. Green lines, trunk; black lines, head; red lines, forelimbs; blue lines, hind limbs. The solid and dotted lines represent right and left limbs, respectively.

We validated the performance of the MCS using sample videos. The sample videos were obtained while a male monkey (body weight: 9 kg) performed shuttling tasks, in which the monkey obtained rewards (pieces of apple) by alternatively visiting two diagonal corners in the cage. To record sample data containing various motions, the following 3 sessions of the shuttling task with different obstacles were conducted (Fig 2A); 1) session 1—without obstacles, 2) session 2—with obstacles (plastic bars through the cage) to induce jumping, and 3) session 3—with obstacles to induce crawling. Each session consisted of 10–13 trials (1 trial = moving from one corner to the other). The 3D video of each of the sessions was analyzed using the present MCS, and the mean processing time (the time needed for the fitting algorithm to reach a steady state), number of manually corrected frames, and total time used for analyzing all the frames were measured to quantify the cost of the analysis. Parameters were measured while the software was run on a PC equipped with a Core i5 750 2.67 GHz processor (4 cores) and 4 GB of random-access memory.

To validate the accuracy of the MCS, positions of the jaw (the center of sphere 1 in Fig 1C), the head (sphere 3), the chest (sphere 6), the abdomen (sphere 8), the elbow (sphere 12), the hand (sphere 16), the knee (sphere 19), and the foot (sphere 22) were estimated using two combined protocols: manual estimation based on visual inspection by two blind experimenters (Experimenter 1 and Experimenter 2) and MCS-assisted estimation by the same two experimenters (MCS-1 and MCS-2). To this end, video frames were randomly selected from each session. In manual estimation, the blind experimenters manually mapped the skeletal model onto the 3D hull in each of the selected frames, while in the MCS-assisted estimation the experimenters performed the same task except now using the aid of the MCS. The positions of the body parts estimated in the four protocols were then compared. In addition, head directions, defined as the directions from the head (sphere 3) to the jaw (sphere 1), were similarly compared. Absolute errors were defined as differences in estimation values between two different estimations (i.e., manual vs. MCS-assisted estimations, or estimations by two different experimenters in the same estimation protocol) in the laboratory coordinate system. In addition, relative errors were defined as differences in estimation values between two different estimations in the coordinate system (abdomen coordinate system) with the origin in the estimated center of the abdomen (sphere 8) according to a previous paper [30].

To validate the effectiveness of the MCS for extracting monkey actions, the following three actions in sessions 2 and 3 were estimated by the two blind experimenters in both the MCS-assisted and manual protocols. Bar-crossing time was defined as the interval from the moment of first crossing the bar by either hand (sphere 16) to the moment of finally crossing the bar by either foot (sphere 22). Jumping time was defined as the period during which both feet (sphere 22) did not touch the floor (height of feet above the floor > 20 cm). Crawling time was defined as the period during which the chest (sphere 6) was near the floor (chest height above floor < 20 cm) and the height of the chest was similar or lower than the height of the abdomen (sphere 8) (the height of the chest–the height of the abdomen < 10 cm). Onset, offset, and duration of each event were estimated in sessions 2 and 3. Then, the absolute and relative errors between two different estimations (manual vs. MCS-assisted estimations, or estimations by two different experimenters in the same estimation protocol) were compared. All data of the position estimation used for this validation and the following experiment are shown in S2 File.

### Effects of methamphetamine (MAP) on spontaneous behaviors

In this experiment, we recorded spontaneous behaviors after administration of saline or MAP in four monkeys (three adult male monkeys, weighing approximately 5 kg and one adult female monkey, weighing approximately 11 kg). The minimal sample size for a paired t-test is n = 4 calculated by a free statistical power analysis program (G*power; http://www.gpower.hhu.de/) [31], in which the minimal sample size was calculated using following parameters; the significance level = 0.05, the effect size = 3.05 based on the data of a previous behavioral experiment on monkeys with amphetamine [32]. MAP was dissolved in saline at a concentration of 5 mg/ml. Monkeys were administrated either MAP (0.5 mg/kg, i.m., at a dose to induce motor dysfunction [32]) or vehicle saline (0.1 ml/kg, i.m.) in its home cage, and were then transferred to the recording cage. Note that, in the previous studies [32, 33] as well as the present study, no signs of suffering were observed in monkeys administered the same dose of MAP. Seven min after the administration, spontaneous behaviors of the monkey were captured by the MCS for 5 min. After at least a 6 day period to washout the drug, the same monkeys were administered the agent that was not administered in the initial recording, and the resulting behaviors were similarly captured. All experiments were performed between 12:00–17:00. One day before each behavioral recording, the monkeys were habituated to the recording cage for 1 hr.

The recorded 3D videos were analyzed with the present MCS, and traces of the body parts of the monkeys were acquired. To quantify motor activity, the following parameters were analyzed: total path length of the monkeys [total length of the trace of the chest (sphere 6 in Fig 1C)], mean walking speed [mean speed of the chest when the monkey was standing], and mean speed of head rotation [speed of the jaw (sphere 1 in Fig 1C) relative to the head (sphere 3 in Fig 1C) when the monkey was crouching]. Since the head also moved during walking, we focused on head rotation while the monkey was crouching. Crouching was defined as such when the height of the hip (i.e., height of the middle point between the left and right sphere 9 in Fig 1C) from the floor of the cage was less than 9 cm, and the speed of the chest was less than 8 cm/sec (this criterion was introduced to eliminate movements in a low posture). Motor activities were compared between saline and MAP using a paired t-test with a significance level of p < 0.05. Furthermore, to check the reproducibility of the results, correlations of the total path length of a monkey, mean walking speed, and mean speed of head rotation between the two experimenters in the MCS-assisted protocol were assessed. Statistical tests were performed using Excel 2013 (Microsoft) and Matlab version R2013b (Mathworks).

## Results

### Comparison of the data between the MCS-assisted and manual estimation

The monkeys show characteristic, but stable behaviors during the shuttling task. Compared with normal walking in session 1 without obstacles, the monkeys showed jumping in session 2 with obstacles in the lower half of the cage and crawling in session 3 with obstacles in the upper half of the cage (Fig 2A). The MCS could trace various motions of the monkey during the task (Fig 2B and 2C). To validate usefulness of the MCS, the estimated data were compared between the MCS-assisted and manual protocols (Table 1). The mean processing time was approximately 30 msec/frame, indicating the fitting algorithm could work in near real-time (Table 1B). Although a substantial number of manual corrections were required, these manual corrections did not significantly increase the analysis time. Since each manual correction could be finished quickly (S1 Movie), the total time used for the analyses was around 5–6 times that the actual duration of the video (Table 1B). The mean estimation errors of the position of body parts were 3–7 cm in the head and the trunk and 4–14 cm in the limbs when estimations performed by Experimenter 1 in the MCS-assisted protocol were compared with those of Experimenter 2 in the manual protocol (Table 1C). The mean error of the head direction ranged from 35° to 43° (Table 1C).

**Table 1:** Video property analyzed (A), estimation cost for the MCS-assisted protocol (B), and absolute estimation errors between the MSC-assisted and manual protocols (C) when Experimenter 1 analyzed the videos in each session with the MCS-assisted protocol while Experimenter 2 analyzed the same videos in the manual protocol.

	Session 1	Session 2	Session 3
*A*. *Video property*			
No. of frames	2216	5509	4343
Total duration (sec)	83.7	206.7	162.4
Frame rate (frames/sec)	26.5	26.7	26.7
No. of events analyzed	13	11	10
*B*. *Cost for the analysis by the MCS*			
Processing time (msec/frame)	33.4 ± 22.5	25.8 ± 24.5	24.9 ± 19.9
No. of manually corrected frames (%)	1.1	1.2	1.2
Total time for analysis (sec)	505.5	1131.0	833.0
*C*. *Absolute estimation errors (MCS-assisted vs*. *manual protocols)*			
Jaw (cm)	6.1 ± 3.0	6.6 ± 3.1	6.2 ± 2.8
Head (cm)	3.3 ± 1.7	4.5 ± 2.4	4.7 ± 3.3
Chest (cm)	3.7 ± 1.7	5.3 ± 2.4	5.0 ± 2.5
Abdomen (cm)	4.0 ± 2.0	5.5 ± 3.4	6.1 ± 3.2
Elbow (cm)	4.8 ± 2.7	7.3 ± 3.9	7.2 ± 4.1
Hand (cm)	7.0 ± 7.2	9.5 ± 7.0	8.6 ± 6.5
Knee (cm)	5.0 ± 3.0	8.6 ± 4.9	9.9 ± 7.4
Foot (cm)	6.3 ± 4.9	13.3 ± 8.9	13.9 ± 9.9
Head direction (degrees)	43.4 ± 16.1	38.3 ± 26.0	35.9 ± 18.0

Processing times and absolute estimation errors are expressed as mean ± SD.

**Fig 3:**
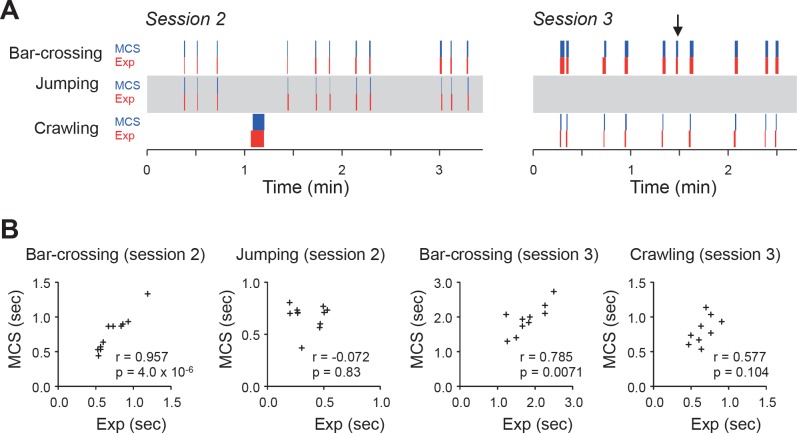
Validation of behavioral event detection by the MCS. A: Chronograms of behavioral events in the MCS-assisted estimation (MCS, blue) and manual estimation based on visual inspection (Exp, red) in session 2 (left) and session 3 (right). Note that there was no false positive nor false negative detection in the chronograms. The monkey displayed crawling once, but did not cross the bars in session 2. In the 6th trial in session 3 (arrow), the monkey crossed the bars without crawling, i.e., it passed between the bars. B: Correlation of the duration of behavioral events between MCS-assisted and manual estimation. Values in each graph indicate the correlation coefficient (r) and p-value of the correlation (p).

Monkey actions (Bar-crossing, Jumping, and Crawling) were estimated by Experimenter 1 in the MCS-assisted protocol and Experimenter 2 in the manual protocol. The chronograms of detected actions indicated that there was no false positive nor false negative detection of events when the experimenter analyzed the video in the MCS-assisted protocol compared with those in the manual protocol (Fig 3A). The durations of the actions analyzed in the MCS-assisted protocol were significantly correlated with those in the manual protocols analyzed (Fig 3B); the duration of bar-crossing in the two protocols were significantly correlated in sessions 2 and 3 (r = 0.957, 0.785, respectively; p < 0.01). However, the correlation between the duration of jumping and crawling in the two protocols were not significant (p > 0.05).

### Reproducibility of estimation using the MCS

To evaluate the reproducibility of position estimation for each body part using the MCS, estimation of the motion data was performed under four different estimation protocols: manual estimation based on visual inspection by Experimenters 1 and 2 (Exp-1, Exp-2) and the MCS-assisted estimation by the same two experimenters (MCS-1, MCS-2). In each comparison of absolute estimation errors in the shuttling task among the four estimation protocols, there was a significant main effect (p < 0.05, repeated measures one-way ANOVA) (S1 Fig). The estimation errors of most body parts in all sessions were significantly smaller when Experimenters 1 and 2 estimated positions of the body parts in the MCS-assisted protocol (MCS1 vs MCS-2; 1–9 cm and 6–20° for the positions of body parts and the head rotation, respectively) (p < 0.05, Bonferroni’s test). The same comparisons by relative errors in the abdomen coordinate system indicated comparable results (S2 Fig), although the magnitudes of the relative errors were slightly (10–20%) larger than the absolute errors.

**Fig 4:**
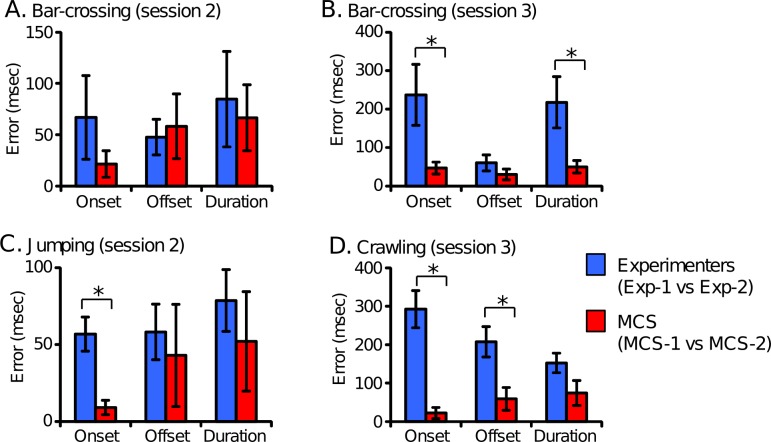
Comparison of behavioral event detection errors between the two different experimenters in MCS-assisted and manual estimation in the shuttling task. Estimation errors of onset and offset timings and duration in the shuttling task were compared. * Significant difference, p < 0.05 (paired t-test). Error bars represent SEMs.

Estimation errors between the two different experimenters was compared between the manual and MCS-assisted protocols (Fig 4). Absolute estimation errors of onset, offset, and duration of the four actions in the shuttling task between two different experimenters were significantly smaller in the MCS-assisted protocol than the manual protocol in five of the 12 parameters (onset of bar-crossing in session 3, manual: 237 ± 79 msec, MCS: 46 ± 15 msec; duration of bar-crossing in session 3, manual: 218 ± 67 msec, MCS: 50 ± 16 msec; onset of jumping, manual: 57 ± 11 msec, MCS: 9 ± 5 msec; onset of crawling, manual: 292 ± 48 msec, MCS: 22 ± 15 msec; offset of crawling, manual: 207 ± 40 msec, MCS: 60 ± 30 msec) (p < 0.05, paired t-test). Furthermore, it should be noted that, in all parameters, the errors were not significantly larger in MCS-assisted estimation than manual estimation. Taken together, these results demonstrate that the MCS can significantly improve the reproducibility of estimation of both body-part positions and detection of actions.

### Effects of methamphetamine on spontaneous behaviors

**Fig 5:**
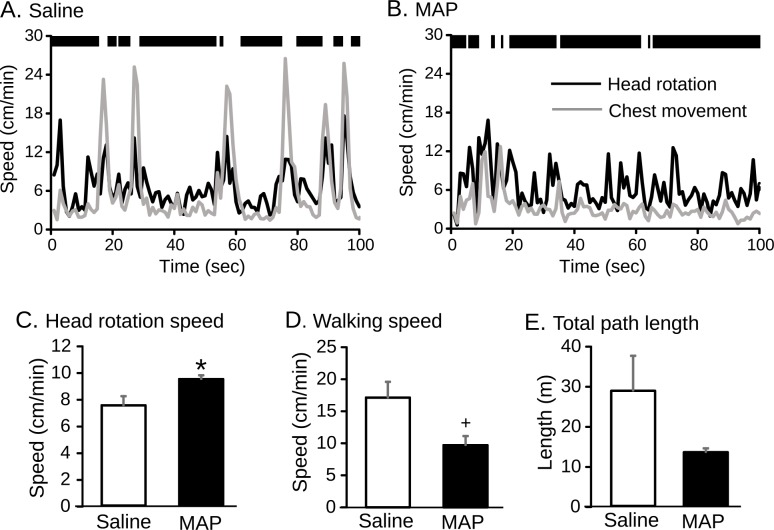
Effects of MAP on spontaneous behaviors. A and B: Examples of the time course of head rotation speed (black line) and chest speed (gray line) of a monkey after administration of saline (A) and MAP (B). Thick black bars above the graph represent periods when the monkey was crouching. C-E: Comparison of motor activities between saline and MAP in the MCS-assisted estimation. * Significant difference, p < 0.05. + Tended toward significance, p < 0.1.

**Fig 6:**
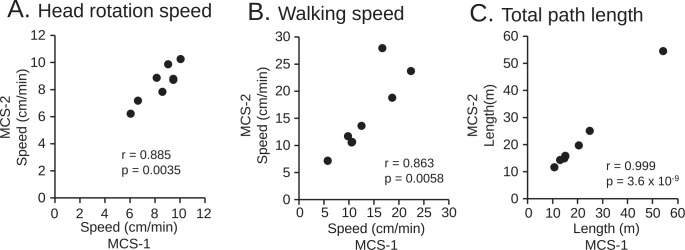
Reproducibility of estimated data using the MCS in the MAP experiment. A-C: Correlation of motor activities in the MAP experiment between the two different experimenters in MCS-assisted estimation.

Head rotation and chest movements were analyzed in the MCS-assisted protocol. Examples of the time courses of head rotation speed (black lines) and the speed of the chest (gray lines) of a monkey after administration of saline (A) and MAP (B) were shown in Fig 5. The mean speed of head rotation was faster in monkeys administered MAP than those given saline, especially while the monkey was crouching. On the other hand, the speed of chest movement while standing (i.e., walking speed) seemed to be slower following MAP administration than saline in this particular monkey. The mean speed of head rotation was significantly increased by MAP (Fig 5C; saline: 7.5 ± 0.7 cm/min; MAP: 9.4 ± 0.3 cm/min; p = 0.038, paired t-test). Mean walking speed tended to be decreased by MAP (Fig 5D; saline: 17.1 ± 2.5 cm/min; MAP: 9.7 ± 1.4 cm/min; p = 0.061, paired t-test). It is of note that there was no significant difference in the mean path length (Fig 5E; saline: 17.1 ± 2.5 cm/min; MAP: 9.7 ± 1, 4 cm/min, p = 0.16, paired t-test). Fig 6 shows correlations of the three parameters of motor activity in the MAP administration experiment as estimated by the two different experimenters in the MCS-assisted protocol (MCS-1 vs. MCS-2). The parameters were highly correlated (r = 0.863 to 0.999, p = 0.0058 to 3.6 × 10^−9^).

## Discussion

### Comparison of MCS-assisted and manual estimation

We developed a novel 3D markerless MCS for monkeys. We have shown that action detection in the MCS-assisted protocol was almost identical to that in the manual protocol (Fig 3A), and that duration estimation of bar-crossing in the MCS-assisted protocol were significantly correlated with estimation by visual inspection (Fig 3B). However, the correlation between the duration of jumping and crawling between the manual and MCS-assisted protocols were not significant. This non-significant results might be ascribed to their low variation relative to the estimation errors. Furthermore, the mean error of body part position between the MCS-assisted and manual estimation was 3–7 cm in the head and trunk and 4–14 cm in the limbs (Table 1C). These errors are not particularly high, given that the height of a monkey was approximately 90 cm, and that the errors between the two experimenters using manual estimation were 3–6 cm in the head and trunk and 3–9 cm in the limbs (S1 Fig). On the other hand, the mean error of head direction ranged from 35° to 43° (Table 1C; S1 Fig). The errors are also comparable to the error between the two experimenters in manual estimation (25–35°, S1 Fig). These findings suggest that MCS-assisted estimation is comparable to manual estimation. Second, we also confirmed that the data estimated by the two experimenters using the MCS-assisted estimation were more consistent than those from manual estimation (S1 Fig; Fig 4). Furthermore, estimations by two different experimenters using the same MCS were highly correlated (Fig 6). These findings suggest that the MCS can contribute to improving the reproducibility of experiments. Third, manual counting of multiple different actions and estimation of different parameters based on visual inspection often requires several playbacks of the same video. However, in the MCS-assisted estimation, once videos are analyzed, any behaviors with a specific definition can be automatically analyzed afterwards. Therefore, MCS-assisted motion capture is very effective for such studies, i.e., analyzing multiple parameters in a video, because once the skeleton model was fitted onto the 3D video at each frame, many behavioral events and parameters can be automatically calculated from the data (e.g., Figs 3 and 5). Fourth, the present MCS can acquire data without markers that could alter behaviors. These findings suggest that the present MCS is useful for analyzing various postures and behaviors of monkeys.

### Comparison with previous MCSs

As far as we know, two studies have proposed systems for markerless whole-body motion capture in monkeys for locomotion analyses. One study [26] focused on the development of an algorithm for a 3D video reconstruction from multiple color videos, requiring manual digitization of each joint position in each frame in the 3D video. The other study [25] implemented a computerized pose estimation algorithm; the estimation error of wrist position was around 2.6 cm, which was smaller than those in the present study. However, because the study focused on movements during locomotion on a treadmill, it is unclear whether their algorithm can be applied to an analysis of a freely moving monkey in a cage, as in the present study. In particular, because they used a statistical method to detect each arm joint in an image, the algorithm may not be robust enough for in-cage behaviors, where the number of possible postures, and views of the arm joints, is remarkably larger compared with that in locomotion on a treadmill. Further tests of their algorithm for in-cage behaviors and integration with the present MCS would be interesting. On the other hand, other sophisticated algorithms have been suggested for human motion capture [34,35], which may also be applicable to monkey behaviors. An important difference between humans and monkeys is the larger amount of fur in monkeys, which makes the surfaces of body parts less clear. Proper modification of the algorithms for humans and their integration into the present MCS could increase the accuracy and robustness of posture estimation.

### Application to neuroscientific research

Analysis using the MCS indicated that acute MAP administration decreased locomotion speed, and did not affect total path length. These findings are inconsistent with rodent studies in which acute MAP administration usually increased locomotor activity [36–38]. However, a previous monkey study reported that the effects of acute MAP administration on motor excitation were not consistent across species [29], which is consistent with the present study. In humans, clinical studies reported that the alteration of motor activity is typically demonstrated by involuntary movements of the face, arms, legs, and trunk, and, at times, movements were rapid and ballistic (see review by Caligiuri and Buitenhuys. [39]). In the present study, the mean head rotation speed was increased, which might correspond to the ballistic movements seen in humans. These findings suggest that hyperkinesia induced by MAP might manifest differently in primates and rodents. On the other hand, a previous study reported that humans feel alert and jittery after MAP administration [40]. The increase in head rotation speed might reflect more cautious behaviors associated with psychological alterations induced by MAP. Further studies are required to determine whether changes in head rotation speed reflect motor excitation or psychological changes. The present results at least suggest the usefulness of the 3D MCS for non-human primates to establish animal models of psychiatric disorders.

As motion capture can provide data with better temporal resolution than visual inspection, it can also be applied to analyses of neural correlation to various behaviors. It is reported that the temporal lobe, including the superior temporal sulcus and extrastriate body area, respond to biological motion and specific body postures [41–43]. Furthermore, inferotemporal cortical neurons encode the medial axis (inner skeleton) of objects as well as surface information [44]. In addition, a psychological study suggests that structural (i.e., axial or skeletal) information of the body is necessary for the recognition of biological motion [45]. These findings suggest that the skeletal information of the body is encoded and represented in the brain. Thus, the present MCS with simultaneous recording of neural activity in the brain may be used to investigate such neural representation of the body skeleton.

### Limitations

There are some limitations in the present MCS that should be improved in future studies. First, a substantial number of manual interventions is required for pose estimation (approximately every 100 frames on average). This lengthens analysis, although the frequent manual intervention had little effect on reproducibility (S1 Fig) because the interventions can be readily performed in a simple way (S1 Movie). The number of manual interventions is larger than those in our previous MCS for rats using similar algorithms [27]. In the previous MCS for rats, the MCS can usually continue estimation for 1 min (1800 frames) without any manual intervention. This difference is ascribed to the higher degrees of freedom of monkey joints and the fact that some body parts are often occluded partially by other body parts in monkeys. Thus, increasing the number of cameras and/or adding some algorithms proposed for humans to compensate for a smaller number of views [34,35] could increase the robustness of pose estimation. Second, the present MCS cannot analyze more than two monkeys. Motion capture of two or more monkeys during social interaction would be important for quantifying social behaviors [5,10,16]. Markerless motion capture of closely interacting subjects is challenging even in the field of human motion capture. Two subjects in the same view remarkably increases degrees of freedom and occlusion and would require more complicated algorithms [46]. However, if the two monkeys are kept separated in different cages to prevent close interaction, the present MCS should work as well as in cases with one subject. Even in such separated situations, monkeys can still demonstrate various social behaviors [10, 47, 48]. Thus, the current MCS could contribute to analyses of social behaviors in monkeys.

## Conclusion

In this paper, we proposed a novel computerized markerless 3D MCS for monkeys freely moving in a cage, for the first time. The results of validation and application to the behavioral pharmacological experiment demonstrated that the present MCS has sufficient accuracy and high reproducibility for quantifying spontaneous behaviors. Thus, the markerless MCS allows analyses of emotional states and social interactions of monkeys, which are easily altered if tracking markers were attached to body parts. These characteristics of the present MCS suggest that the present MCS would be useful for translational pre-clinical research in drug development using monkeys for various psychiatric disorders that display behavioral abnormalities.

## Supporting Information

S1 Fig Comparison of the absolute errors in sessions 1 (A), session 2 (B), and session 3 (C). MCS-1 and MCS-2 indicate data from MCS-assisted estimation by the Experimenters 1 and 2, respectively. Exp-1 and Exp-2 indicate data from manual estimation by Experimenters 1 and 2, respectively. * Significant difference from the absolute errors between MCS-1 and MCS-2 (Bonferroni’s test, p < 0.05). Error bars represent SEMs. A number below each bar graph indicates number of samples in each group.
(EPS)

S2 Fig Comparison of the relative errors in the abdomen coordinate system. The descriptions as for S1 Fig. Note that the errors of the abdomen are not shown since the abdomen is the origin of the abdomen coordinate system, and that the errors of the head direction are not shown since the angles estimations were same regardless of the coordinate systems.
(EPS)

S1 File Software for the pose estimation using a 3D video, its source codes, and a set of sample data files. (ZIP)

S2 File All position estimation data used in the present analyses. (ZIP)

S1 Movie A demonstration showing how the position estimation process was performed with the present MCS-assisted system. (MP4)
