# Supplementary material for: A Markerless 3D Computerized Motion Capture System Incorporating a Skeleton Model for Monkeys
Source: PLoS One. 2016 Nov 3;11(11):e0166154. doi: 10.1371/journal.pone.0166154 (PMC5094601; doi:10.1371/journal.pone.0166154)
Supplement: S2 File — (ZIP) [file pone.0166154.s004.zip › File S2/About Files.pdf]

The files in this directory are raw position estimation data used in the present paper.

Each txt file corresponds with an experimental session indicated by its file name.

The format of the file name is as follows:

[name of experiment]- [subject ID]-[experimental session / drug]- Experimenter ID estimated]-[method of estimation].txt

In a file, each line represents data of a certain video frame. The description for each column in a line is as follows:

Column 1:	timestamp of the frame
Column 2-4:	xyz coordinate of a part of head (1 in Fig.1C)
Column 5-7:	xyz coordinate of a part of head (2 in Fig.1C)
Column 8-10:	xyz coordinate of a part of head (3in Fig.1C)
Column 11-13:	xyz coordinate of a part of head (4 in Fig.1C)
Column 14-16:	xyz coordinate of neck (5 in Fig.1C)
Column 17-19:	xyz coordinate of a part of trunk (6 in Fig.1C)
Column 20-22:	xyz coordinate of a part of trunk (7 in Fig.1C)
Column 23-25:	xyz coordinate a part of trunk (8 in Fig.1C)
Column 26-28:	xyz coordinate of a part of left forelimb (10 in Fig.1C)
Column 29-31:	xyz coordinate of a part of left forelimb (11 in Fig.1C)
Column 32-34:	xyz coordinate of a part of left forelimb (12 in Fig.1C)
Column 35-37:	xyz coordinate of a part of left forelimb (13 in Fig.1C)
Column 38-40:	xyz coordinate of a part of left forelimb (14 in Fig.1C)
Column 41-43:	xyz coordinate of a part of left forelimb (15 in Fig.1C)
Column 44-46:	xyz coordinate of a part of left forelimb (16 in Fig.1C)
Column 47-49:	xyz coordinate of a part of right forelimb (10 in Fig.1C)

Column 50-52:	xyz coordinate of a part of right forelimb (11 in Fig.1C)
Column 53-55:	xyz coordinate of a part of right forelimb (12 in Fig.1C)
Column 56-58:	xyz coordinate of a part of right forelimb (13 in Fig.1C)
Column 59-61:	xyz coordinate of a part of right forelimb (14 in Fig.1C)
Column 62-64:	xyz coordinate of a part of right forelimb (15 in Fig.1C)
Column 65-67:	xyz coordinate of a part of right forelimb (16 in Fig.1C)
Column 68-70:	xyz coordinate of a part of left hind limb (9 in Fig.1C)
Column 71-73:	xyz coordinate of a part of left hind limb (17 in Fig.1C)
Column 74-76:	xyz coordinate of a part of left hind limb (18 in Fig.1C)
Column 77-79:	xyz coordinate of a part of left hind limb (19 in Fig.1C)
Column 80-82:	xyz coordinate of a part of left hind limb (20 in Fig.1C)
Column 83-85:	xyz coordinate of a part of left hind limb (21 in Fig.1C)
Column 86-88:	xyz coordinate of a part of left hind limb (22 in Fig.1C)
Column 89-91:	xyz coordinate of a part of right hind limb (9 in Fig.1C)
Column 92-94:	xyz coordinate of a part of right hind limb (17 in Fig.1C)
Column 95-97:	xyz coordinate of a part of right hind limb (18 in Fig.1C)
Column 98-100:	xyz coordinate of a part of right hind limb (19 in Fig.1C)
Column 101-103:	xyz coordinate of a part of right hind limb (20 in Fig.1C)
Column 104-106:	xyz coordinate of a part of right hind limb (21 in Fig.1C)
Column 107-109:	xyz coordinate of a part of right hind limb (22 in Fig.1C)
Column 110-112:	xyz coordinate of tale (23 in Fig.1C)

*Note that these coordinates are those before smoothing with loess filter.
